# Comparison of Serum Mitochondrial Open Reading Frame of the 12S rRNA-c (MOTS-c) Levels in Patients With Multiple Sclerosis and Healthy Controls

**DOI:** 10.7759/cureus.26981

**Published:** 2022-07-18

**Authors:** Selma Tekin, Levent Sinan Bir, Esin Avci, Hande Şenol, Işık Tekin, Ufuk Çınkır

**Affiliations:** 1 Department of Neurology, Pamukkale University School of Medicine, Denizli, TUR; 2 Department of Medical Biochemistry, Pamukkale University School of Medicine, Denizli, TUR; 3 Department of Biostatistics, Pamukkale University School of Medicine, Denizli, TUR; 4 Department of Cardiology, Pamukkale University School of Medicine, Denizli, TUR; 5 Department of Neurology, Istanbul Basaksehir Cam and Sakura State Hospital, Denizli, TUR

**Keywords:** hypertension, hyperlipidemia, diabetes mellitus, homa-ir, ms patients, mots-c, multiple sclerosis

## Abstract

Background

Multiple sclerosis (MS) is a major global problem, and as its pathogenesis is understood more clearly, therapeutic options expand accordingly. The mitochondrial open reading frame of the 12S rRNA-c (MOTS-c) is a novel mitochondria-derived protein acting on metabolic homeostasis. In this study, we aimed to investigate the role of serum MOTS-c in the pathophysiology of the disease in MS patients and to discuss the mechanism of MOTS-c.

Methodology

In total, 43 patients diagnosed with relapsing-remitting MS and 41 healthy controls were enrolled in the study. MOTS-c, fasting blood glucose, insulin, Homeostatic Model Assessment for Insulin Resistance (HOMA-IR), lipid panel, and body mass index levels were assessed.

Results

The participants’ MOTS-c levels remained significantly lower than that of the control group, while their fasting blood glucose and HOMA-IR values were higher. The multivariate logistic regression analysis established that increased MOTS-c levels could be a protective factor against the development of MS disease. The area under the receiver operating characteristic curve for MOTS-c was calculated as 0.782 (95% confidence interval = 0.684-0.879, p = 0.0001).

Conclusions

This study is the first to scrutinize MOTS-c levels in MS patients. We tried to provide clinical evidence that MOTS-c could act as a highly discriminative biomarker between MS patients and control groups, which may hold great promise for future therapeutic options.

## Introduction

Multiple sclerosis (MS), an immune-mediated, demyelinating, and neurodegenerative disease, is more prevalent among women, often among young adults. While the pathogenesis of MS is still full of mysteries, low vitamin D levels, Epstein-Barr virus infection, smoking, exposure to ultraviolet B light, and obesity are risk factors responsible for its pathogenesis [1]. In addition to these environmental factors, genetic background is also a major determinant of MS development.

It has been well-documented that diabetes mellitus (DM), cardiovascular diseases, and vascular disease risk factors, such as hyperlipidemia and hypertension (HT), which are more prevalent in MS patients, adversely affect the clinical progression of MS [2,3]. Published clinical trials have reported that childhood and adolescent obesity might increase the risk of MS development and progression [4,5].

Autoreactive T-lymphocytes, B-lymphocytes, and macrophages with the activation of microglial cells, various antibodies, complements and cytokines, which result in mitochondrial dysfunction, presence of metalloproteinases, and oxidative stress are all among the underlying mechanisms responsible for neuronal and axonal damage of the brain and spinal cord in MS [6].

Insulin resistance (IR) is a chronic inflammatory process aggravating neuroinflammation linked to diseases, such as MS [7]. IR and oxidative stress are closely interrelated within a vicious cycle. Multiple lines of evidence suggest that increased secretion of pro-inflammatory cytokines induced by oxidative stress can promote IR, which also induces oxidative stress [8]. IR and oxidative stress have been shown to be more prevalent among MS patients than in controls, both of which exacerbate the progression of the disease in these patients [9].

The mitochondrial open reading frame of the 12S rRNA-c (MOTS-c) is a novel mitochondria-encoded peptide in circulation and is involved in some endocrine activities. MOTS-c, found in tissues such as muscle, brain, and liver, can be measured in blood plasma and cerebrospinal fluid [10]. Previous studies have revealed that MOTS-c levels are associated with IR and obesity markers including body mass index (BMI), waist circumference, waist-to-hip ratio, fasting insulin level, Homeostatic Model Assessment for Insulin Resistance (HOMA-IR), and HbA1c [11].

MOTS-c has the potential to yield numerous metabolic benefits. The clinical experiments performed so far have confirmed that these effects have some positive association with AMP-activated protein kinase (AMPK) activity, elevated endogenous 5-aminoimidazole-4-carboxamide-1-β-d-ribofuranoside (AICAR) levels, and inhibition of oxidative phosphorylation while enhancing glucose utilization and fatty acid oxidation. MOTS-c may also accelerate muscle glucose uptake in response to insulin production and modulate metabolites to regulate insulin sensitivity in mice fed with a high-fat diet, thereby promoting insulin sensitivity [10].

As identified by recent clinical research, there is a clear association between MOTS-c and obstructive sleep apnea syndrome, polycystic ovary syndrome, and vascular risk factors, such as endothelial dysfunction [12-14]. In this context, our research aimed to further explore the current knowledge of MOTS-c levels in MS disease, whose pathogenesis has not been fully elucidated and which is associated with many metabolic abnormalities. Thus, we hoped to contribute to the literature through our findings to shed new light on both the pathogenesis and treatment of MS.

## Materials and methods

Study design and participants

In total, 41 healthy volunteers and 43 patients diagnosed with relapsing-remitting MS aged 18-75 years who presented to the neurology department of a tertiary-level hospital were enrolled in our study. Neurological examinations of all the patients were performed prospectively, and their demographic data and the expanded disease severity scale (EDSS) scores were recorded. Exclusion criteria included the presence of other accompanying neurodegenerative disorders, chronic alcohol and drug abuse, and progressive MS diagnosis. Informed consent forms were obtained from all the enrolled participants. The study was conducted following the guidelines recommended by the Medical Ethics Committee of Pamukkale University dated 02.03.2021 and numbered 05.

Study procedure

Initially, approximately 5 mL of venous blood was drawn into biochemistry tubes containing SST gel from the patient and the control group following at least an eight-hour fast in the morning. The serum specimens obtained after centrifugation at 3,500 rpm for 10 minutes were transferred into Eppendorf tubes and stored at -80 degrees until the trial day. MOTS-c levels were analyzed from the prepared serum at room temperature on the trial day in the research laboratory of the Department of Medical Biochemistry using a commercial competitive enzyme-linked immunosorbent assay kit (IT Laboratory-Shanghai, China).

After the standards and chemicals of the kits used in the study were prepared, the standards and serum samples were pipetted into the wells in the microplate. By following the steps described in the instructions, the serum samples were colored based on the concentrations of the tests. After color formation was observed, the absorbance values of the wells were read at 450 nm using a Biotek Elx800 Microplate reader (BioTek Instruments Inc., Winooski, VA, USA). The concentrations were calculated using the serum absorbance values on the Gen5 data analysis program. The resulting values were expressed as ng/mL.

Statistical analysis

All statistical analyses were performed using Statistical Package for the Social Sciences (SPSS) version 25 (IBM Corp., Armonk, NY, USA). The continuous variables were reported as mean ± standard deviation and the categorical variables as number and percentage. The normality of the data was checked using the Shapiro-Wilk test. Independent group comparisons were calculated using the Mann-Whitney U test, and a Spearman correlation analysis was performed to investigate the relationships between continuous variables. Univariate and multiple logistic regression analysis was used to identify which variables triggered the development of MS. The difference between the categorical variables was analyzed using the chi-square test. Receiver operating characteristic (ROC) analysis was conducted to identify the presence of MS and optimal cut-off value for MOTS-c levels. Statistical significance was set at p-values of <0.05.

## Results

In total, 43 patients diagnosed with relapsing-remitting MS and 41 healthy volunteers with no history of neurological disorders were included. The mean age of the patients and the controls was 37.47 ± 10.41 and 38.05 ± 10.87, respectively. No significant difference was noted between the patient and the control group in terms of age and gender (p > 0.05).

The patient population included 33 (76.7%) females and 10 (23.3%) males, while the control group consisted of 31 (75.6%) females and 10 (24.4%) males. The mean BMI scores of the patients and the controls corresponded to 25.32 ± 5.41 and 25.88 ± 4.54, respectively, which did not reveal a significant difference (p > 0.05).

Regarding comorbid conditions, eight (18.6%) patients were diagnosed with DM, six (14.0%) with hyperlipidemia, and three (7%) with HT, yet only the presence of DM was significantly higher in patients than in controls (p = 0.03). In addition, the mean EDSS score and the number of attacks in the patient group were 2.21 ± 1.28 and 3.84 ± 2.24, respectively. Thirteen (30.2%) patients were receiving immunomodulatory therapy, 12 (27.9%) glatiramer acetate therapy, eight (19.6%) oral first-line treatment (teriflunomide and dimethyl fumarate), and 10 (23.3%) fingolimod treatment. Table 1 summarizes the clinical and demographic information of the study participants.

**Table 1:** Demographic and clinical findings of study participants. *p < 0.05 is statistically significant. MS: multiple sclerosis; BMI: body mass index; EDSS: expanded disease severity scale

		MS (n = 43)	Control (n = 41)	P-value
Age		38.05 ± 10.87	37.47 ± 10.41	0.971
Gender	male / female	10 (23.3%)/33 (76.7%)	10 (24.4%)/31 (75.6%)	0.903
BMI		25.32 ± 5.41	25.88 ± 4.54	0.388
Diabetes mellitus	-/+	35 (81.4%)/8 (18.6%)	40 (97.6%)/1 (2.4%)	0.003*
Hypertension	-/+	40 (93%)/3 (7%)	38 (92.7%)/3 (7.3%)	1
Hyperlipidemia	-/+	37 (86%)/6 (14%)	35 (85.4%)/6 (14.6%)	0.929
Cardiac disease	-/+	43 (100%)/0 (0%)	41 (100%)/0 (0%)	-
EDSS		2.21 ± 1.28		
Treatment types	Immunmodulator	13 (30.2%)		
	Glatiramer acetate	12 (27.9%)		
	Dymethyl-fumarate	4 (9.3%)		
	Teriflunomide	4 (9.3%)		
	Fingolimode	10 (23.3%)		

When the laboratory data of both cohorts were compared, MOTS-C (p = 0.0001), fasting blood glucose (p = 0.034), and HOMA-IR values (p = 0.027) were observed to differ significantly. The mean MOTS-c level of the patients was 159.23 ± 40.79, which was significantly lower than that of the controls (189.95 ± 22.51, p = 0.0001). The mean values for fasting blood glucose and HOMA-IR in patients were identified as 105.6 ± 30.56 and 3.58 ± 2.82, respectively, both of which were significantly higher than controls (p = 0.034 and p = 0.027, respectively). On the other hand, no significant difference was evident between patients and controls regarding blood lipids, folic acid, and vitamin B12 levels (p > 0.05). Table 2 provides an overview of the laboratory data of study participants.

**Table 2:** The laboratory findings of MS patients and control groups *p < 0.05 is statistically significant. MS: multiple sclerosis; HOMA-IR: Homeostatic Model Assessment Indicator of Insulin Resistance; HDL: high-density cholesterol; LDL: low-density cholesterol; VLDL: very low-density cholesterol; MOTS-c: mitochondrial open reading frame of the 12S rRNA-c

	MS (n = 43)	Control (n = 41)	P-value
	Mean ± SD	Mean ± SD	
Glucose (mg/dL)	105.6 ± 30.56	92.34 ± 8.82	0.034*
Insulin (mU/L)	13.46 ± 10.06	9.03 ± 3.98	0.093
HOMA-IR	3.58 ± 2.82	2.08 ± 1.02	0.027*
Non-HDL cholesterol (mmol/L)	138.28 ± 39.12	128.44 ± 32.79	0.594
LDL cholesterol (mmol/L)	109.3 ± 30.26	102.59 ± 25.9	0.754
HDL cholesterol (mmol/L)	50.26 ± 11.69	53.29 ± 10.48	0.202
Total cholesterol (mmol/L)	187.28 ± 36.66	179.2 ± 31.93	0.71
Triglycerides (mmol/L)	138.14 ± 64.68	123.71 ± 69.46	0.263
VLDL cholesterol (mmol/L)	27.72 ± 13	25.41 ± 13.76	0.383
Folic acid (ug/L)	8.33 ± 3.39	8.73 ± 3.6	0.658
Vitamine B12 (ng/L)	393.76 ± 208.47	358.64 ± 103.86	0.904
MOTS-c	159.23 ± 40.79	189.95 ± 22.51	0.0001*

However, a positive correlation was obtained between MOTS-C values and those of non-high-density cholesterol (HDL) (r = 0.423, p = 0.006), very low-density cholesterol (VLDL) (r = 0.048, p = 0.003), triglyceride (r = 0.489, p = 0.001), and total cholesterol (r = 0.369) (p = 0.018) in the control group (Table 3).

**Table 3:** The significant correlation between MOTS-c and lipid profile in the control group. *p < 0.05 is statistically significant. non-HDL: non-high-density cholesterol; VLDL: very low-density cholesterol; TG: triglycerides; MOTS-c: mitochondrial open-reading-frame of the 12S rRNA-c

	MOTS-c
Non-HDL	p = 0.006*; r = 0.423
VLDL	p = 0.003*; r = 0.448
TG	p = 0.001*; r = 0.489
Total cholesterol	p = 0.018*; r = 0.369

In addition, no correlation was observed between MOTS-c and folic acid and vitamin B12 levels in both cohorts (p > 0.05). No significant difference was found between males and females in relation to MOTS-c values in both groups (p > 0.05). A multiple logistic regression model was developed based on factors with a significant effect on the presence of MS. As is evident from this model, the increase in MOTS-c was a protective factor against MS, independent of DM presence, and HOMA-IR effect (p = 0.002; OR = 0.957) (Table 4).

**Table 4:** Univariate and multiple logistic regression analysis results. *p < 0.05 is statistically significant. OR: odds ratio; CI: confidence interval; DM: diabetes mellitus; HOMA-IR, Homeostatic Model Assessment Indicator of Insulin Resistance; MOTS-c: mitochondrial open reading frame of the 12S rRNA-c

		Wald	P-value	OR	95% CI for OR	
					Lower	Upper
Multiple	DM	1.085	0.298	0.256	0.02	3.326
	HOMA-IR	3.482	0.062	1.362	0.985	1.884
	MOTS-c	9.969	0.002*	0.957	0.932	0.984

**Figure 1:**
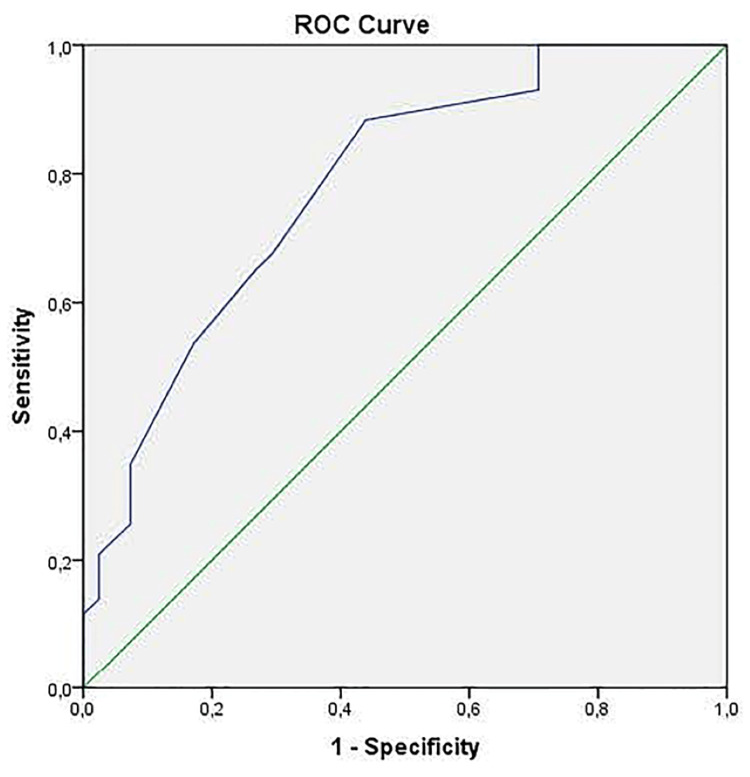
ROC curve analysis for MOTS-c results. ROC: receiver operating characteristic; MOTS-c: mitochondrial open reading frame of the 12S rRNA-c

Regarding the discrimination of MOTS-c values for MS, the area under the ROC curve (AUC) was 0.782 (95% CI = 0.684-0.879, p = 0.0001). Accordingly, if the optimum cut-off point defined according to Youden Index values was set as 190.5, the presence of MS disease could be predicted at 88.4% sensitivity and 56.1% specificity (Figure 1).

## Discussion

The most important clinically relevant finding in our study is significantly lower MOTS-c values in MS patients than in the controls. DM, obesity, hyperlipidemia, and vascular comorbidities, such as coronary artery disease, are more prevalent among MS patients. These comorbid conditions are assumed to act on MS pathogenesis and may further exacerbate disability when accompanying MS [2,3]. The interaction between MOTS-c values and some disorders is well-documented, with previous research indicating that MOTS-c values remain low in patients with obesity, IR, DM, and endothelial dysfunction [14-16]. Consistent with the literature, our results also reveal that MOTS-c values were lower in MS patients than in healthy controls. The lack of a significant difference between both cohorts regarding age, gender, and BMI shows that MOTS-c could be a discriminating indicator in MS, independent of the above-mentioned variables.

MOTS-c is a 16 amino acid peptide synthesized from the mitochondrial 12S rRNA region [10]. Mitochondria constitute a fundamental system that maintains metabolic functions by regulating cellular energy homeostasis. However, mitochondrial dysfunction triggers deficiency in the electron transport chain, beta-oxidation, and associated IR [16]. A clinical trial on rodents in relation to the metabolic regulation effects of MOTS-c suggested that the activation of a mechanism linked to AMPK enhances glucose utilization in skeletal muscle, improving insulin sensitivity and accelerating restoration of metabolic homeostasis [17]. The chronic inflammatory process in MS induces mitochondrial DNA mutations through the production of reactive oxygen species, eventually resulting in mitochondrial damage. This metabolic stress triggers protein misfolding in the endoplasmic reticulum, disruptions in energy production, neuronal losses, and ultimately neuroaxonal dysfunction [6]. All these processes in close connection to mitochondrial dysfunction can account for lower MOTS-c levels in MS noted in our study.

The findings we obtained from our clinical trials established that fasting blood glucose and HOMA-IR values as well as the presence of DM were significantly higher in MS patients than in healthy controls. MS increases the risk of developing type 1 DM and increases the incidence of type 2 DM compared to the general population [7]. This is assumed to emerge because of the IR triggered by cytokines which arise due to MS-induced autoimmune processes and chronic inflammation. Previous reports have also documented that IR is likely to worsen disability in MS patients [9]. In addition, our univariate analysis revealed that DM, fasting blood glucose, insulin, HOMA-IR, and MOTS-c were significant contributors to MS development. Insulin abnormalities may also enhance inflammatory response and oxidative stress [18]. Peripheral hyperinsulinemia leads to increased insulin secretion in the cerebrospinal fluid. Paradoxically, persistent hyperinsulinemia downregulates insulin receptors at the blood-brain barrier, reducing insulin delivery to the brain. Thus, IR-associated peripheral hyperinsulinemia brings about hypoinsulinemia in the central nervous system, which impairs both cognition and selective brain function [7]. In relation to the protective effect of insulin on the brain, neurodegenerative diseases, such as MS, may arise because of decreased central insulin.

Higher HOMA-IR values in our patient group with low MOTS-c values compared to the controls confirm the results reported by previous studies highlighting the interaction between MOTS-c and IR [11,12]. A substantial body of experimental and clinical research lends support to the interplay between plasma MOTS-c concentrations and increased insulin sensitivity [10,15,19]. Overexpression of MOTS-c enhances glucose uptake in myoblasts. A study conducted on mice concluded that intraperitoneal intervention of MOTS-c could regulate age and diet-dependent IR by activating the AMPK pathway in skeletal muscle and enhancing GLUT4 expression [17]. The impact of MOTS-c on increasing insulin sensitivity is well-established, but as our multivariate analysis reveals, increased MOTS-c level may exert a protective effect on MS development once DM and HOMA-IR factors are excluded. This finding suggests that MOTS-c is involved in the pathogenesis of MS in a different fashion.

Our results did not yield a significant difference between both cohorts concerning BMI values. Previous research did not report a marked difference between MS patients and healthy individuals regarding BMI but found higher BMI values in MS patients with IR than in individuals without IR [9]. They attributed this to the fact that oxidative stress in the pathogenesis of MS is not triggered by weight alone but also by other underlying mechanisms and may contribute to weight gain.

While we did not observe a strong relationship between MOTS-c values ​​and blood lipids in our patient cohort, a significant positive correlation was noted between MOTS-c and non-HDL, VLDL, TG, and total cholesterol levels ​​in the controls. Published research also reported a positive correlation between circulating MOTS-c and LDL and total cholesterol levels [17]. In addition, increased lipid production enhances MOTS-c expression while insulin inhibits lipid-induced MOTS-c [13]. Lower IR in our control cohort was not a significant contributor to the relationship between MOTS-c ​​and lipids. By contrast, the higher IR in our patients tended to suppress the lipid-induced MOTS-c expression, blocking the interaction between MOTS-c and lipids. In fact, this critical difference is suggestive of the involvement of different processes affecting the working mechanism of MOTS-c in MS patients.

While some studies suggest that MOTS-c interacts with the folate-methionine cycle and inhibits folate production [10], others report that MOTS-c does not target the folate cycle [19]. Similarly, we did not identify a significant interplay between MOTS-c and folate levels in our study.

Healthy skeletal muscles have metabolic flexibility that can change glucose and fat utilization on an as-needed basis. Dodecanedioic acid (DAC), a dicarboxylic acid, acts as an alternative energy source when metabolic flexibility is lost [20]. MOTS-c has been reported to boost energy capacity [21], reduce muscular fatigue, and improve physical performance by increasing DAC uptake in skeletal muscle during exercise in obese mice [19]. Fatigue is one of the prevalent clinical manifestations of MS that profoundly degrades the quality of life. It should be noted that low MOTS-c levels and fatigue might be interrelated at the molecular level, and MOTS-c treatment can potentially eliminate this symptom. The insights provided so far into the pathogenesis and course of MS have helped to make a tremendous advancement in MS treatment. However, current treatments provide only partial protection against neurodegenerative components of MS. Further research on the merit of early treatment and powerful agents is important for evidence-based approaches to treating and managing MS [22]. Clinical trials on the utilization of MOTS-c as a treatment tool have yielded very promising results thus far [23].

Similar to prior studies [11,17], our study did not detect any significant difference between genders in MOTS-c levels. Although some research noted a difference in the obese male children relative to the controls, no difference existed between obese males and obese females [11].

In our ROC analysis, we found that when the area under the ROC curve of MOTS-c was 0.782, its sensitivity was high, although specificity was low at this value. This indicates that MOTS-c might act as an effective biomarker in diagnosing MS but falls short of distinguishing the healthy group.

Limitations

A major limitation of this study lies in the fact that our patient cohort consisted of only relapsing-remitting MS patients, leading us not to evaluate the course of MOTS-c in progressive patients. Second, the fact that EDSS was not at a very advanced stage restricted our evaluation of the impact of MOTS-c on advanced disability. MOTS-c levels should be addressed in a larger and clinically diverse patient population in future studies.

## Conclusions

We attempted to provide clinical evidence that MOTS-c could be a highly discriminative biomarker between MS patients and controls, which might hold great promise for new therapeutic options. In addition, the finding that overexpression of MOTS-c can be a protective factor against MS development after excluding DM and HOMA-IR effect is suggestive of its involvement in MS pathogenesis through different mechanisms. There is a need for further studies to be planned experimentally in the future to confirm that, with its effect on MS pathogenesis, MOTS-c can be protective against fatigue, attack, and progression.

## References

[1] Pantazou V, Schluep M, Du Pasquier R (2015). Environmental factors in multiple sclerosis. Presse Med.

[2] Sicras-Mainar A, Ruíz-Beato E, Navarro-Artieda R, Maurino J (2017). Comorbidity and metabolic syndrome in patients with multiple sclerosis from Asturias and Catalonia, Spain. BMC Neurol.

[3] Marrie RA (2019). Comorbidity in multiple sclerosis: past, present and future. Clin Invest Med.

[4] Munger KL, Chitnis T, Ascherio A (2009). Body size and risk of MS in two cohorts of US women. Neurology.

[5] Ben-Zacharia AB (2018). The effects of body mass index (BMI) on multiple sclerosis (MS) progression. Ann Mult Scler Res.

[6] Dendrou CA, Fugger L, Friese MA (2015). Immunopathology of multiple sclerosis. Nat Rev Immunol.

[7] Watson GS, Craft S (2006). Insulin resistance, inflammation, and cognition in Alzheimer's disease: lessons for multiple sclerosis. J Neurol Sci.

[8] Styskal J, Van Remmen H, Richardson A, Salmon AB (2012). Oxidative stress and diabetes: what can we learn about insulin resistance from antioxidant mutant mouse models?. Free Radic Biol Med.

[9] Oliveira SR, Simão AN, Kallaur AP (2014). Disability in patients with multiple sclerosis: influence of insulin resistance, adiposity, and oxidative stress. Nutrition.

[10] Lee C, Zeng J, Drew BG (2015). The mitochondrial-derived peptide MOTS-c promotes metabolic homeostasis and reduces obesity and insulin resistance. Cell Metab.

[11] Du C, Zhang C, Wu W (2018). Circulating MOTS-c levels are decreased in obese male children and adolescents and associated with insulin resistance. Pediatr Diabetes.

[12] Baylan FA, Yarar E (2021). Relationship between the mitochondria-derived peptide MOTS-c and insulin resistance in obstructive sleep apnea. Sleep Breath.

[13] Ramanjaneya M, Jerobin J, Bettahi I (2019). Lipids and insulin regulate mitochondrial-derived peptide (MOTS-c) in PCOS and healthy subjects. Clin Endocrinol (Oxf).

[14] Qin Q, Delrio S, Wan J, Jay Widmer R, Cohen P, Lerman LO, Lerman A (2018). Downregulation of circulating MOTS-c levels in patients with coronary endothelial dysfunction. Int J Cardiol.

[15] Cataldo LR, Fernández-Verdejo R, Santos JL, Galgani JE (2018). Plasma MOTS-c levels are associated with insulin sensitivity in lean but not in obese individuals. J Investig Med.

[16] Lu H, Koshkin V, Allister EM, Gyulkhandanyan AV, Wheeler MB (2010). Molecular and metabolic evidence for mitochondrial defects associated with beta-cell dysfunction in a mouse model of type 2 diabetes. Diabetes.

[17] Ramanjaneya M, Bettahi I, Jerobin J (2019). Mitochondrial-derived peptides are down regulated in diabetes subjects. Front Endocrinol (Lausanne).

[18] Krogh-Madsen R, Plomgaard P, Keller P, Keller C, Pedersen BK (2004). Insulin stimulates interleukin-6 and tumor necrosis factor-alpha gene expression in human subcutaneous adipose tissue. Am J Physiol Endocrinol Metab.

[19] Kim SJ, Miller B, Mehta HH (2019). The mitochondrial-derived peptide MOTS-c is a regulator of plasma metabolites and enhances insulin sensitivity. Physiol Rep.

[20] Salinari S, Bertuzzi A, Gandolfi A, Greco AV, Scarfone A, Manco M, Mingrone G (2006). Dodecanedioic acid overcomes metabolic inflexibility in type 2 diabetic subjects. Am J Physiol Endocrinol Metab.

[21] Lee C (2019). Nuclear transcriptional regulation by mitochondrial-encoded MOTS-c. Mol Cell Oncol.

[22] Hauser SL, Cree BA (2020). Treatment of multiple sclerosis: a review. Am J Med.

[23] Wei M, Gan L, Liu Z (2020). Mitochondrial-derived peptide MOTS-c attenuates vascular calcification and secondary myocardial remodeling via adenosine monophosphate-activated protein kinase signaling pathway. Cardiorenal Med.

